# Cefazolin Concentration in the Mediastinal Adipose Tissue of Patients Undergoing Cardiac Surgery

**DOI:** 10.21470/1678-9741-2016-0031

**Published:** 2017

**Authors:** Rodrigo Mezzalira Tchaick, Michel Pompeu Barros Oliveira Sá, Fernando Ribeiro de Moraes Figueira, Kilma Coelho Paz, Álvaro Antonio Bandeira Ferraz, Fernando Ribeiro de Moraes Neto

**Affiliations:** 1 Division of Cardiovascular Surgery, Hospital Dom Helder Câmara (HDH), Cabo de Santo Agostinho, PE, Brazil; 2 Division of Cardiovascular Surgery, Pronto-Socorro Cardiológico de Pernambuco (PROCAPE), Recife, PE, Brazil; 3 Division of Cardiovascular Surgery, Instituto de Medicina Integral Professor Fernando Figueira Ringgold, Recife, PE, Brazil; 4 Laboratório de Imunopatologia Keizo Asami (LIKA), Universidade Federal de Pernambuco (UFPE), Recife, PE, Brazil; 5 Universidade Federal de Pernambuco (UFPE), Recife, PE, Brazil

**Keywords:** Cefazolin, Cardiac Surgical Procedures, Adipose Tissue, Mediastinum

## Abstract

**Objective:**

To measure the concentration of cefazolin in the anterior mediastinal adipose tissue of patients undergoing cardiac surgery, determining the variation of cefazolin concentration.

**Methods:**

Two samples of approximately 1g of subcutaneous tissue were collected from 19 patients who underwent surgery in December 2015: the first sample was collected right after sternotomy and the second one, before sternal synthesis with steel wires. Antibiotic dosage was administered through high performance liquid chromatography.

**Results:**

We observed a positive and statistically significant correlation between time 1 and cefazolin concentration (r=0.489 and *P*=0.039). For time 2 and cefazolin concentration, there was a negative and statistically significant correlation between both variables (r=-0.793 and *P*<0.001). A negative correlation was also observed between body mass index and cefazolin concentration at time 2 (r=-0.510 and *P*=0.031). The regression model showed that every 1-minute increase in time 1 corresponded to an increase of 0.240 µg/dL in cefazolin concentration, whereas every 1-minute increase in time 2 corresponded to a reduction of 0.046 µg/dL in cefazolin concentration. As for body mass index, every 1 kg/m^2^ increase corresponded to a reduction of about 0.510 µg/dL in cefazolin concentration.

**Conclusion:**

There was a positive and significant correlation between the initial time of surgery and cefazolin level in the first dosage. The evaluation of the second dosage showed a negative and significant correlation between cefazolin level and the second time of dosage. The concentration of cefazolin is under the influence of body mass index.

**Table:** 

Abbreviations, acronyms & symbols	
AUC	= Area under the curve
BMI	= Body mass index
CABG	= Coronary artery bypass grafting
CPB	= Cardiopulmonary bypass
DSWI	= Deep sternal wound infections
HPLC	= High performance liquid chromatography
IMA	= Internal mammary artery
LIKA	= Laboratório de Imunopatologia Keizo Asami
MIC	= Minimum inhibitory concentration
SPSS	= Statistical Package for Social Sciences

## INTRODUCTION

Mediastinitis is a serious infectious complication in the postoperative period of cardiovascular surgery^[1]^. The overall incidence of deep sternal wound infections (DSWI) ranges from 0.4% to 5%. Despite the low incidence, damage to patients and hospital costs are substantial^[2]^.

Antibiotic prophylaxis is one of the main preventive measures. The importance of prophylactic antibiotics for cardiac surgery has been widely studied in Brazil by Sá et al.^[2]^, who evaluated several studies carried out over the last 30 years. The use of different antibiotics to the same end clearly indicates that there is no consensus on the efficacy and safety of each one^[3]^. Therefore, there is still no absolute certainty that the main antibiotics used effectively reach the tissues where infection occurs.

The objective of this study is to measure the concentration of cefazolin in the adipose tissue of the mediastinum in patients undergoing cardiac surgery in order to determine variation in cefazolin concentrations at the beginning and at the end of surgery.

## METHODS

### Location and Period of the Study

This study was conducted with 19 patients undergoing cardiac surgery at Dom Helder Câmara Hospital in Cabo de Santo Agostinho, Pernambuco, Brazil. The patients were operated in December 2015 and their summarized profile is presented in Table 1.

**Table 1:** Characteristics of the population.

Characteristics	n = 19 (%)
Age (years)	
Mean (SD)	60.3 (8.2)
Minimum - Maximum	30 - 75
Hypertension	16 (84.21%)
Diabetes	9 (47.36%)
Dyslipidemia	11 (57.8%)
Smoking	7 (36.8%)
CABG	14 (73.68%)
Valve surgery	4 (21.05%)
Aortic aneurysm	1 (5.26%)

SD=standard deviation; CABG=coronary artery bypass graft surgery

### Ethical Aspects

This research was approved by the Research Ethics Committee of the Health Sciences Center of Federal University of Pernambuco. This research follows the principles of the Helsinki Declaration for research on humans.

### Inclusion Criteria

Patients over 18 years old who underwent cardiac surgery with cardiopulmonary bypass (CPB) [coronary artery bypass grafting (CABG) surgery, valve replacement, aortic aneurysm or multiple cardiac surgery] in which the access to the heart was through the sternum (sternotomy).

### Exclusion Criteria

Patients requiring antibiotic prophylaxis other than cefazolin; patients with impaired renal function (creatinine > 1.5 mg/dl); patients who had low preoperative cardiac output with the use of vasoactive drugs.

### Technical Procedures

All patients received an antibiotic regimen with cefazolin administered as follows: a first dose of 2g diluted in 100 ml saline at anesthesia induction, followed by a dose of 1g diluted in 100 mL saline, every four hours during surgery. The cefazolin used in all surgical procedures had the same trademark. The company which produces the antibiotic played no part in this study, so that there was no conflict of interest.

Regarding the minimum inhibitory concentration (MIC), the following recommendations were considered: for *Staphylococcus aureus,* 1 µg/mL, and for *Staphylococcus epidermidis*, 4 µg/mL^[4,5]^.

### Sample Preparation

Two samples of approximately 1 g of subcutaneous tissue were collected for analysis: the first sample was collected right after sternotomy (time 1) and the second, before sternal synthesis with steel wires, near the end of surgery (time 2). The tissue was collected from the anterior mediastinum. The samples were shipped in a styrofoam box with ice, at a temperature of -80ºC to Laboratório de Imunopatologia Keizo Asami (LIKA) and were thawed only at the time of analysis.

Samples were prepared according to the protocol described by Sings et al.^[6]^, in 1984, and more recently reproduced by Waltrip et al.^[7]^. The protocol is as follows:

A) weigh the tissue sample on a precision scale;
B) mix the sample to a cooled extraction solution containing 70% methanol and 30% 0.1M sodium acetate (pH 5.2) in a volume ratio of 1:2 (*e.g*., 1g of tissue to 1.11 mL solution, considering the density of adipose tissue as 0.9 g/cc);
C) homogenize the sample for 30 seconds;
D) cool it at -20ºC for ten minutes;
E) centrifuge it at 15000 rpm for 15 minutes;
F) collect the supernatant and centrifuge it again for 15 minutes at 15000 rpm; and
G) filter it using a high performance liquid chromatography (HPLC) 0.22 µm filter.

### Sample Analysis

The measuring of the antibiotic dose in the samples was performed by HPLC reverse phase with the ÄKTA Purifier 10 GE^®^ system. The column used was C18-300, 250 x 4.6 mm ID. A specialized biochemist carried out this analysis.

The mobile phase consisted of 85% 0.01M sodium acetate (pH 5.2) and 15% of a solution comprised of 96% acetonitrile and 4% methanol. A volume of 100 µl of the solution was injected through a C18-300, 250 x 4.6 mm, column with a flow rate of 1.5 ml/min through an isocratic method.

Cefazolin absorbency was measured at a 254 nm wavelength using an ultraviolet detector device. Chromatography was performed at room temperature. For each sample, three analyses were performed separately. A standard curve for cefazolin was established through the adipose tissue samples, which were initially free of cefazolin and then received the antibiotic in increasing concentrations of 10, 20, 30, 40 and 50 µg/ml. The chromatograms of the samples and the areas corresponding to the cefazolin curve were drawn by Unicorn 4.11^®^ software, which received the data directly from the ÄKTA purifier machine 10.

### Statistical Analysis

To analyze the data, first a database with patient information was built using a simple table in Microsoft Office Excel^®^. The data was then exported to Statistical Package for Social Sciences (SPSS) version 18, whereby the statistical analysis was performed. The qualitative variables were: hypertension, diabetes, dyslipidemia, smoking, CABG surgery, valve surgery, or correction of aortic aneurysm. They were described in absolute and relative frequency (percentage). Quantitative variables, such as body mass index (BMI), time of first and second collections, and first and second level of cefazolin, were expressed as minimum, maximum, mean, median, and standard deviation. In addition, the confidence interval for each variable was calculated.

Normal distribution was assessed using the Kolmogorov-Smirnov test. To assess the correlations "time 1 x cefazolin level 1", "time 2 x cefazolin level 2", and "BMI x cefazolin level 2", the Pearson's correlation coefficient was used, where a normal behavior for the distribution of variables was observed. Additionally, a linear regression model adjustment (Spearman's test) was applied in order to determine the degree of increase or decrease in cefazolin levels from the variation in the time of data collection and the patient's BMI. All statistical conclusions were drawn considering a significance level of 5%.

## RESULTS

In total, 19 patients were analyzed. Average age of patients was 60.3 years and most individuals were female (63.2%) (Table 1). The BMI analysis found 15 (79.8%) patients with BMI <30 kg/m^2^, 3 (15.8%) patients had a BMI between 30-40 kg/m^2^, and only 1 (5.26%) was higher than 40 kg/m^2^.

With regard to cefazolin concentration, time 1 average was 6.10 µg/mL (SD 2.2 µg/mL; 4.45-10.86). For time 2, the average was 8.40 µg/mL (SD 2.9 µg/mL; 4.04-13.02). The concentration of cefazolin showed a normal distribution (*P*=0.507 and *P*=0.878, respectively) for both times.

As for the time of measurement, on average, the group had a mean time of 26:30 minutes (SD 4:30; 19-35) at time 1. At time 2, the mean time was 180 minutes (SD 49.9; 60-210). It was observed that the distribution of time 1 and time 2 showed normal behavior (*P*=0.827 and *P*=0.148, respectively).

Table 2 brings a descriptive analysis of patients evaluated according to BMI, time 1, time 2, and cefazolin concentrations at times 1 and 2. Mean BMI was 26.5 kg/m^2^ (SD 4.1). The normality test was not significant (*P*=0.999), indicating that the distribution of BMI in the group evaluated presented normal behavior.

**Table 2:** Analysis of the measures.

Measures	Min - Max	Mean	SD	Median	CI	*P*-value^1^
BMI	18.7 - 33.30	26.5	4.1	26.6	24.5 - 28.6	0.999
Time 1	19 - 35	26.5	4.5	26.0	23.8 - 28.2	0.827
Time 2	60 - 210	180	49.9	151.9	127.1 - 176.7	0.148
Cefazolin 1	4.4 - 10.9	6.8	2.2	6.1	5.7 - 7.9	0.507
Cefazolin 2	4.0 - 13	8.1	2.9	8.4	6.6 - 9.5	0.878

1 *P*-value of Kolmogorov-Smirnov test (if *P*-value <0.05; the measure assessed does not have normal distribution). BMI=body mass index; SD=standard deviation; CI=confidence interval

Table 3 shows the analysis of the correlation between the measurements taken and the adjustment of the regression model between them. It is noteworthy that a positive and statistically significant correlation was found between time 1 and cefazolin concentration at time 1 (r=0.489 and *P*=0.039), indicating that an increase in time 1 is proportionately followed by an increase in cefazolin concentration at time 1. The evaluation of time 2 and cefazolin concentration at time 2 showed a negative and statistically significant correlation between both variables (r=-0.793 and *P*<0.001), indicating that an increase in time 2 is followed by a reduction in cefazolin concentration at time 2. A negative correlation between BMI and cefazolin concentration at time 2 was also observed (r=-0.510 and *P*=0.031), which means that an increase in BMI is followed by a decrease in cefazolin concentration at time 2.

**Table 3:** Correlation analysis and adjustment of the regression model between the evaluated measures.

	Correlation		Regression		
	r	*P*-value^(*)^	b	r^2^	*P*-value^(**)^
Time 1 x cefazolin 1	0.489	0.039	0.239	0.239	0.039
Time 2 x cefazolin 2	-0.793	< 0.001	-0.046	0.628	< 0.001
BMI x cefazolin 2	-0.510	0.031	-0.366	0.259	0.031

r=Pearson's coefficient; b= coefficients of the logistic equation; r^2^=strength of the association

(*) *P*-value regarding Pearson's coefficient test.

(**) *P*-value regarding Spearman's coefficient test

**Fig. 1:**
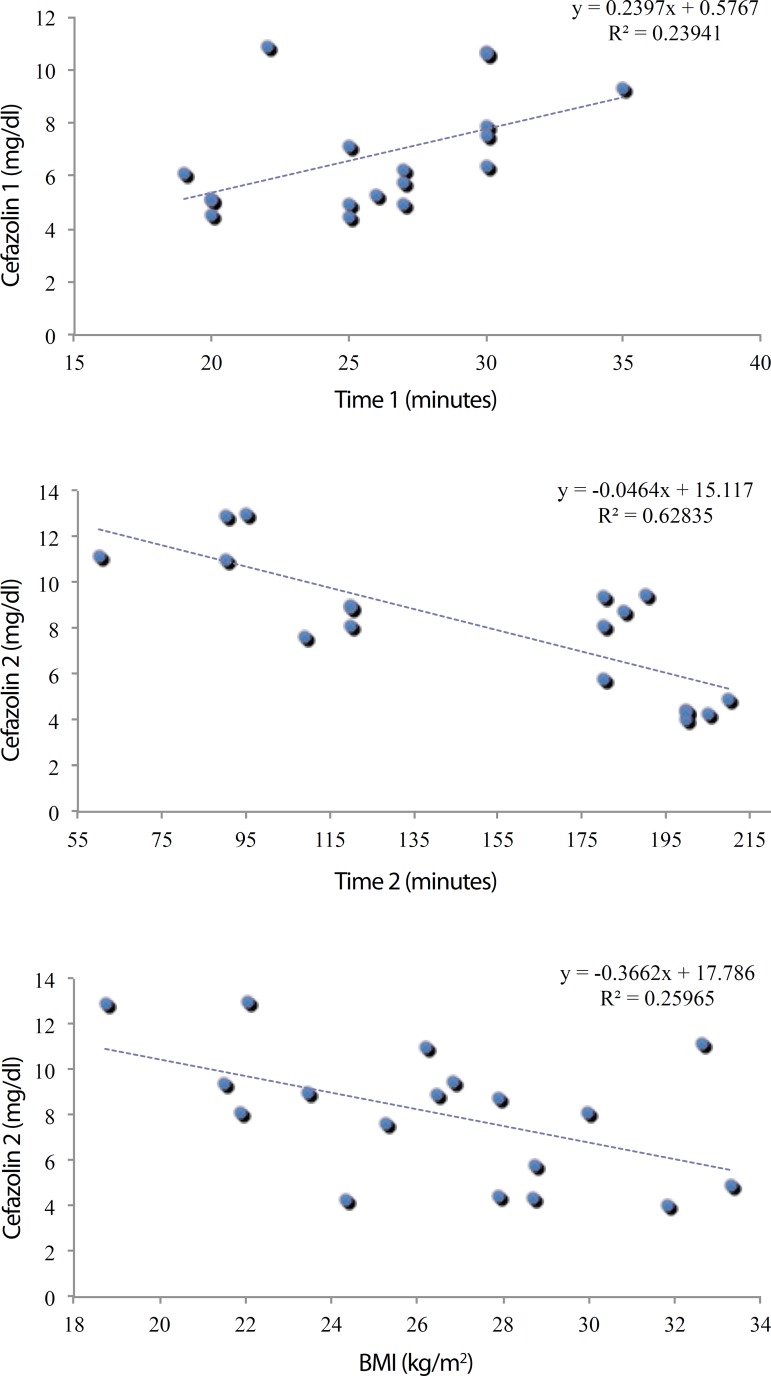
Adjustment of the regression models.

The regression model showed that every one-minute increase in time 1 corresponded, on average, to an increase of 0.240 µg/dL in the cefazolin concentration (Figure 1A), whereas every one-minute increase in time 2 corresponded to a reduction of 0.046 µg/dL in the cefazolin concentration (Figure 1B). As for BMI, every 1 kg/m^2^ increase corresponded to a reduction of about 0.510 µg/dL in cefazolin concentration at time 2 (Figure 1C).

## DISCUSSION

To validate the HPCL method in the analysis of cephalosporins, Sings et al.^[6]^ described the liquid chromatography analysis of 10 different cephalosporins using an extraction procedure with easily adjustable reagents for each cefazolin, in any of its three mobile phases. All experiments were performed with human plasma collected from the Akron City Hospital blood bank, which had antibiotics added to it. The chromatography was performed at room temperature using ultraviolet detection at 254 nm and a C-18 column (300 x 4 mm). Antibiotics were quantified by measuring the peak heights of each cephalosporin, which were determined by the characteristics of each substance^[6]^.

The statistical analysis of the chromatographic data for each plasma sample supplemented with antibiotic was conducted in all therapeutic ranges of each cephalosporin. These various therapeutic ranges were determined from clinical data collected in previous studies, using the microbiological assay agar diffusion. Following the aforementioned analysis, the study stated that the HPLC method was in accordance with the clinical acceptability criteria, with excellent precision, specificity, and sensitivity. In this sense, data from the study indicate that HPLC is easily adaptable to the dosage of cephalosporins levels.

Thus, this study corroborates what is now recommended in the Brazilian and American Pharmacopoeias regarding the quantification of cefazolin, which confirms the reliability of the results found here, for very similar methods of diluents were used and, with respect to the column (C-18300, 250 x 4.6 mm), we applied a more advanced technique which provides more accurate results^[6]^.

Toothaker et al.^[8]^ published a review article on analytical methods for cephalosporins in biological fluids and showed that, since 1980, chromatography has been the primary technique used for the analysis of cephalosporins measurements. This technology provides selectivity, accuracy, as well as ease of use. In the studied articles, it was also noted that the proper selection of the method depends on the constraints imposed by the objective of the study. The studies evaluated cephalosporins measured in the muscle tissue, bone, and urine, and, in most of them, HPLC was the preferred technique since it has the ability to distinguish between the drug and other metabolites^[8]^.

Pevzner et al.^[9]^ assayed cefazolin in adipose tissue of 29 patients by agar diffusion in an attempt to assess its distribution according to BMI. Two grams of this antibiotic were injected 30-60 minutes before incision. Their results showed that cefazolin concentrations were inversely related to BMI and a considerable number of patients with a BMI above 40 did not reach minimum inhibitory concentrations for Gram negative cocci, suggesting that the currently recommended dose may result in failure in obese patients. This study indicates that, in patients with BMI below 40, a 2g dose of cefazolin is sufficient to reach the minimum inhibitory concentration, which is consistent with the results found in the sample of our study, even though we used a different technique from that recommended by the Brazilian and American pharmacopoeias.

Waltrip et al.^[7]^ carried out a study aiming to assess the safety of the prophylaxis with cefazolin schemes in cardiac surgery. Adipose tissue samples of the subcutaneous tissue of 34 patients were collected near the sternal wound, and then taken to the laboratory to establish the cefazolin concentrations, which were determined by the HPLC method. The results showed that, in group 1 (receiving 1 g of cefazolin preoperatively and 1 g at the end of the procedure), the concentration of cefazolin ranged from 3 to 6 µg/mL and, in group 2 (given 2 g of cefazolin preoperatively and continuous infusion of 20 mg/min throughout the surgery), it ranged from 5 to 16 µg/mL. It was noted then that, in group 1, the safe concentration was not maintained throughout the surgery whereas, in group 2 (which received a further similar scheme to that used in the work herein), the minimum inhibitory concentration was achieved throughout the whole surgical procedure.

Fellinger et al.^[10]^ analyzed the serum concentration of cefazolin in patients who underwent cardiac surgery with CPB. A group of 10 patients received 1 gram of cefazolin during anesthetic induction and 1 gram right before CPB. The cefazolin was extracted from blood samples and measured by HPLC 5 times. Cefazolin levels remained consistently above MIC_90_ for *S. aureus* as well as for *S. epidermidis* throughout the time of surgery. The highest seric levels of cefazolin, with an average of 35.56 µg/mL, occurred after the second dose of cefazolin during CPB. The lowest seric levels of cefazolin, averaging 6.34 µg/mL, were found right after the start of CPB and before administration of the second dose of cefazolin. It is noticeable that a satisfactory dose of cefazolin serum may be correlated with effective doses in the adipose tissue of the mediastinum.

Caffarelli et al.^[11]^ sought to assess the effects of CPB and profound hypothermic circulatory arrest on plasma cefazolin levels administered for antimicrobial prophylaxis in cardiovascular surgery. Four groups (10 patients per group) were prospectively studied: vascular surgery without CPB (group A), cardiac surgery with a CPB time of less than 120 minutes (group B), cardiac surgery with a CPB time of greater than 120 minutes (group C), and cardiac surgery with CPB and profound hypothermic circulatory arrest (group D). Subjects received cefazolin at induction and before wound closure. Arterial blood samples were obtained preceding cefazolin administration, at skin incision, hourly during the operation, and before redosing. Cefazolin plasma concentrations were determined by radial diffusion assay, with *Staphylococcus aureus* as the indicator microorganism. Cefazolin plasma concentrations were considered noninhibitory at 8 µg/mL or less, intermediate at 16 µg/mL, and inhibitory at 32 µg/mL or greater. In group A, cefazolin plasma concentrations remained greater than 16 µg/mL during the complete surgical procedure. In group B, cefazolin plasma concentrations diminished to 16 µg/mL or less in 30% of the patients, but remained above 8 µg/mL. In group C, cefazolin plasma concentrations decreased to less than 16 µg/mL in 60% of patients and were less than 8 µg/mL in 50% of patients. In group D, cefazolin plasma concentrations reached 16 µg/mL in 66% of the patients and decreased to 8 µg/mL in only 1 patient. The authors concluded that for patients undergoing cardiac surgery with a CPB time of greater than 120 minutes, a single dose of cefazolin before skin incision with redosing at wound closure does not provide targeted antimicrobial cefazolin plasma levels during the entire surgical procedure.

Considering that routine use of the internal mammary artery (IMA) as a bypass graft increases the risk of DSWI, possibly by a surgically induced perfusion deficit^[12]^, Andreas et al.^[13]^ hypothesized that IMA preparation impairs antibiotic penetration into presternal tissue during CABG. The authors carried out a study with eight patients undergoing skeletonized left IMA harvesting for CABG. Standard antibiotic prophylaxis was administered: 4g of cefazolin before skin incision and an additional 2g during skin closure. Concentrations of cefazolin were measured in subcutaneous tissue on the presternal right and left sides (surgically affected) after sternotomy and, additionally, in subcutaneous tissue on the thigh (surgically unaffected) by microdialysis over a 10-hour period. In the results, mean peak tissue concentration and the area under the curve (AUC) on the left sternal side were significantly reduced compared with the right side and the thigh (mean peak concentration, 13.1±5.8 *versus* 24.1±4.7 and 27.8±9.7 µg/mL; *P*=0.005 and *P*=0.013; AUC 74.2±31.0 *versus* 110.4±25.0 and 140.3±46.3 µg x hours per milliliter; *P*=0.004 and *P*=0.002). Mean subcutaneous concentrations of cefazolin on the left sternal side exceeded the minimal inhibitory concentration (MIC_90_) of *Staphylococcus epidermidis* of 4 g/mL in only 37.5% of the patients after 5 hours. The authors concluded that IMA harvesting significantly impairs local antibiotic penetration during CABG and common antibiotic dosing schemas should be reevaluated in this cardiac surgical setting. Even with the protection failing in 3 patients, the overall average of the antibiotic concentration was maintained throughout the surgery at high levels (17.5 µg/ ml), well above those found in the study carried out here. It must be considered that the prophylactic dose used (4g at the start of surgery and 2g at the end) was twice that used in the sample presented here. Moreover, even though Andreas et al.^[13]^ collected the cefazolin through microdialysis, the measurement was performed through the HPLC method.

## CONCLUSION

An appropriate concentration of cefazolin was found in the adipose tissue of the mediastinum of the studied population, both at the beginning of the surgery and at its end. There was a positive and significant correlation between the initial time of surgery and cefazolin level in the first dosage. The evaluation of the final time showed a negative and significant correlation between cefazolin level and the second dosage.

**Table:** 

Authors' roles & responsibilities	
RMT	Conception and design of the work; acquisition, analysis, interpretation of data for the work; drafting the work and revising it critically for important intellectual content; final approval of the version to be published
MPBOS	Interpretation of data for the work; drafting the work or revising it critically for important intellectual content; final approval of the version to be published
FAMSF	Acquisition, analysis, interpretation of data for the work; final approval of the version to be published
KCP	Acquisition, analysis, interpretation of data for the work; final approval of the version to be published
AABF	Conception or design of the work; revising it critically for important intellectual content; final approval of the version to be published
FMN	Conception or design of the work; revising it critically for important intellectual content; final approval of the version to be published
